# Maximum Isometric and Dynamic Strength of Mixed Martial Arts Athletes According to Weight Class and Competitive Level

**DOI:** 10.3390/ijerph19148741

**Published:** 2022-07-18

**Authors:** Orlando Folhes, Víctor Machado Reis, Diogo Luís Marques, Henrique Pereira Neiva, Mário Cardoso Marques

**Affiliations:** 1 Department of Sport Sciences, University of Beira Interior, 6201-001 Covilhã, Portugal; orlandofolhes@gmail.com (O.F.); diogo.marques@ubi.pt (D.L.M.); hpn@ubi.pt (H.P.N.); 2 Department of Sport Sciences, Exercise and Health, University of Trás-os-Montes e Alto Douro, 5001-801 Vila Real, Portugal; victormachadoreis@gmail.com; 3 Research Center in Sports Sciences, Health Sciences and Human Development (CIDESD), 6201-001 Covilhã, Portugal

**Keywords:** combat sports, elite athletes, professional athletes, mixed martial arts, heavyweight, lightweight, physical performance, isometric strength, dynamic strength

## Abstract

Mixed martial arts (MMA) athletes must achieve high strength levels to face the physical demands of an MMA fight. This study compared MMA athletes’ maximal isometric and dynamic strength according to the competitive level and weight class. Twenty-one male MMA athletes were divided into lightweight professional (LWP; *n* = 9), lightweight elite (LWE; *n* = 4), heavyweight professional (HWP; *n* = 4), and heavyweight elite (HWE; *n* = 4). The handgrip and isometric lumbar strength tests assessed the isometric strength, and the one-repetition maximum (1RM) bench press and 4RM leg press the dynamic strength. Univariate ANOVA showed differences between groups in absolute and relative 1RM bench press and absolute isometric lumbar strength. Post hoc tests showed differences in 1RM bench press between HWE and LWE (117.0 ± 17.8 kg vs. 81.0 ± 10.0 kg) and HWE and LWP athletes (117.0 ± 17.8 kg vs. 76.7 ± 13.7 kg; 1.5 ± 0.2 kg·BW^−1^ vs. 1.1 ± 0.2 kg·BW^−1^). In addition, there was a correlation between 1RM bench press and isometric lumbar strength for absolute (*r* = 0.67) and relative values (*r* = 0.50). This study showed that the 1RM bench press and isometric lumbar strength were associated and could differentiate MMA athletes according to their competitive level and weight class. Therefore, optimizing the force production in the upper body and lower back seems important in elite and professional MMA athletes.

## 1. Introduction

Mixed martial arts (MMA) is one of the fastest-growing modalities worldwide, being a mixture of combat sports, including judo, boxing, wrestling, Brazilian jiu-jitsu, muay Thai, taekwondo, and karate, among others [1,2,3,4,5]. MMA combat occurs in standing, grappling, percussion, and on the ground [2,3,4,6,7]. During combat, traumatic blows such as punches, elbows, knees, and kicks are valid, as well as immobilization, joint keys, and strangulations, in addition to various projection and control techniques [2,3,4,6,7]. Therefore, strength, a prominent characteristic in MMA, manifests differently during combat.

Even though MMA combat is characterized by intermittent periods of high-intensity muscular actions interspersed by short periods of recovery [1,4,8,9], the fight strategy adopted by the athlete will determine the predominance of force manifestations during its course [8]. For example, grappling maneuvers require expressions of isometric strength, which is considered a critical physical quality for distinguishing elite grapplers [8]. This requirement is noticed when athletes struggle to maintain the best posture and domain in a particular position, especially those that lead the opponent to submission by strangulation, braces, immobilizations, or twists [2,10]. On the other hand, striking techniques (i.e., punching, kicking, elbowing, kneeing, counterstriking, and escaping difficult situations while standing and on the ground) require more dynamic actions than isometric ones, with a particular emphasis on high-velocity actions [1,2,8,11].

Previous research highlighted the need for high levels of dynamic and isometric strength (upper and lower limbs) to improve the performance of critical skills in MMA athletes [1,2,3,8,12,13,14]. For example, James et al. [14] studied male semi-professional and amateur MMA athletes to determine whether maximal strength and other physical performance parameters differed according to competition level (lower-level vs. higher-level competitor). The results showed that higher-level MMA athletes presented significantly higher relative 1RM back squat strength than lower-level athletes (1.8 ± 0.2 kg·BW^−1^ vs. 1.6 ± 0.2 kg·BW^−1^, respectively). In addition, higher-level MMA athletes tended to present higher relative 1RM bench press strength than lower-level athletes (1.2 ± 0.2 kg·BW^−1^ vs. 1.1 ± 0.2 kg·BW^−1^, respectively), although this difference did not reach statistical significance [14]. Therefore, based on these data, the authors suggested that maximal lower-limb dynamic strength is crucial for distinguishing higher-level from lower-level MMA competitors.

Nevertheless, since the James et al. [14] study was conducted with semi-professional and amateur MMA athletes, it is vital to understand whether these results are transferable to professional and elite contexts. While success depends not only on these physical attributes, awareness of these factors is extremely important because it helps to identify the patterns needed to succeed in MMA, particularly at the elite level [8,15]. Therefore, understanding the athletes’ maximum isometric and dynamic strength levels can be crucial for designing specific strength training programs in MMA. In addition, it will contribute to verifying whether isometric and dynamic strength levels can differentiate professional and elite MMA athletes in terms of competitive level and weight class.

Based on the previous premises, we hypothesized that MMA athletes fighting in higher competitive levels and weight classes would present higher isometric and dynamic strength values than those competing in lower competitive levels and weight classes. Therefore, to explore this hypothesis, the current cross-sectional study aimed to compare MMA athletes’ maximum isometric and dynamic strength levels according to the competitive level (elite vs. professional) and weight class (lightweight vs. heavyweight). In addition, we examined whether isometric and dynamic strength variables (upper and lower limbs) establish a relationship to identify which physical attributes are crucial for optimization in MMA athletes.

## 2. Materials and Methods

### 2.1. Study Design

For two weeks, professional and elite Brazilian MMA athletes participated in the current study. The athletes’ strength levels were assessed on two occasions in a laboratory, with a 72 h interval between sessions. On the first day, we measured the body weight (Filizola^®^ scale, PL, São Paulo, Brazil) and height (SANNY, São Paulo, Brazil), followed by the isometric handgrip strength and isometric lumbar strength. The 1RM bench press and 4RM leg press were measured on the second day. Although all athletes were experienced with all exercises due to their strength training routines (training experience of more than five years), we instructed them regarding the testing procedures one week before the evaluation tests to minimize technical errors. In addition, we asked all athletes to maintain their regular hydration and nutrition routines and avoid strenuous exercise 72 h before the evaluation sessions.

### 2.2. Participants

Twenty-one Brazilian male MMA athletes were divided into four groups according to their competitive level and weight class: lightweight professional (LWP; *n* = 9; 30.5 ± 6.2 years), lightweight elite (LWE; *n* = 4; 25.3 ± 1.9 years), heavyweight professional (HWP; *n* = 4; 28.3 ± 3.9 years), and heavyweight elite (HWE; *n* = 4; 29.1 ± 6.0 years). The athletes had more than ten years of combat experience and were considered professional when they participated in more than three professional MMA fights in events accredited by the “Comissão Atlética Brasileira de MMA” (CABMMA), including the UFC, BELLATOR, ONE FC, and SHOOTO. Briefly, UFC, BELLATOR, and ONE FC consist of five 5-min rounds for championship fights and three 5-min rounds for non-title bouts. SHOOTO pro-fights consist of three 5-min rounds, while semi-pros fights consist of two 5-min rounds. If the athletes were ranked among the top ten in their categories based on these events, they were rated as elite MMA athletes. Athletes weighing ≤ 76 kg were considered lightweight, and those weighing > 76 were considered heavyweight, according to CABMMA unified rules [16]. Table 1 presents the athletes’ characteristics according to their competitive level and weight class. Before starting the study, we fully informed the coaches and athletes about the possible risks of the tests. The Ethical Committee of the University approved this study (No. 20/2022), and all athletes signed a written informed consent according to the Declaration of Helsinki.

### 2.3. Procedures

Before the tests, all athletes performed a 10-min warm-up of 5 min of pedaling at a self-selected pace on a stationary bicycle, followed by 5 min of joint mobilization. Two strength and conditioning coaches supervised all testing procedures.

#### 2.3.1. Isometric Handgrip Strength Test

The athletes performed the test in a seated position, with the shoulder slightly adducted, elbow flexed at 90°, forearm in a neutral position, and the wrist between 0° and 30°. After verbal command, we instructed the athletes to squeeze the dynamometer (Jamar TBW^®^, São Paulo, Brazil) as hard as possible. They performed three attempts with both hands and rested 15 s between trials. Then, we registered the maximum value (measured in kilogram-force, kgf) for further analysis. In addition, we summed the maximum values obtained in each hand to create a composite score and the relative values by dividing the absolute value by the body weight (kgf·BW^−1^) [17].

#### 2.3.2. Isometric Lumbar Strength Test

**Figure 1:**
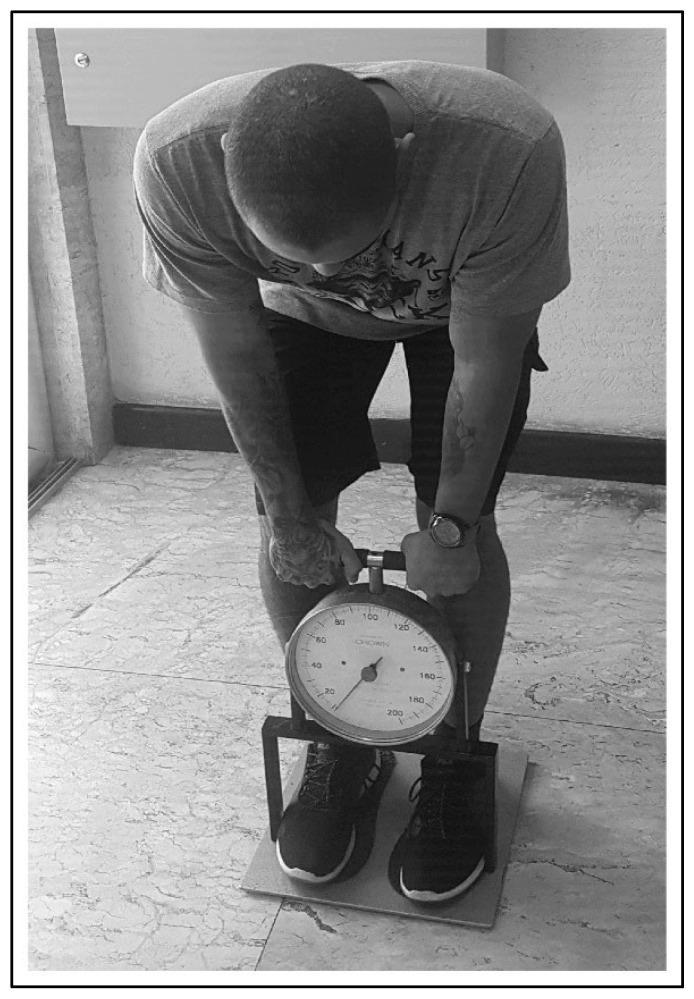
Illustration of the isometric lumbar strength test.

We asked the participants to stand on a platform with the knees semi-flexed, the trunk flexed at an angle of 120°, and the elbows fully extended (Figure 1) [18]. Then, they were asked to perform a maximal isometric trunk extension while grabbing the dynamometer with both hands (Crown Dorsal Dynamometer, Filizola, São Paulo, Brazil), which can measure 200 kgf. All athletes performed three maximal attempts, and we registered the best attempt for further analysis. We calculated the isometric lumbar strength by dividing the absolute value by the body weight (kgf·BW^−1^).

#### 2.3.3. One-Repetition Maximum Bench Press Test

All athletes performed a specific warm-up of 8 repetitions at 50% 1RM, followed by 3 repetitions at 70% 1RM. Then, we increased the weight by 0.4 to 5 kg until the athletes could perform a single lift correctly. We conceded a maximum of 3 attempts to attain the 1RM [19]. The athletes lay down on a flat bench, keeping the scapulae neutral, knees flexed at 90°, and feet flat on the ground. They grasped a 20 kg Olympic barbell with a pronated grip wider than shoulder-width apart. We marked the grip position on the barbell to allow the athletes to adopt the exact grip in the subsequent attempts. The athletes started the test with their elbows fully extended. Then, they descended the barbell in a controlled manner until it touched the chest, and then they performed a concentric action until the elbows were fully extended [19]. Two spotters, one on each side, supervised all procedures. We calculated the relative 1RM bench press by dividing the absolute value by the body weight (kg·BW^−1^).

#### 2.3.4. Four-Repetition Maximum Leg Press Test

All athletes performed a specific warm-up of 8 repetitions at 50% 1RM and 3 repetitions at 70% 1RM. Then, we increased the weight by 1 to 10 kg until the athletes could perform no more than four repetitions correctly. We conceded a maximum of 3 attempts to attain the 4RM. The athletes performed the test in a 45° inclined leg press machine (Pure Leg Press, Technogym, Cesena, Italy). They were seated on the bench with the knees fully extended and feet shoulder-width apart. After instruction, they descended the platform in a controlled manner until a 90° knee flexion, and then they performed a concentric action by extending the knees [20]. Two spotters, one on each side, supervised all procedures. We calculated the relative 4RM leg press by dividing the absolute value by the body weight (kg·BW^−1^).

### 2.4. Statistical Analysis

The Shapiro–Wilk and Levene’s tests checked the assumptions of normality and homogeneity of the variances, respectively. Considering that all variables followed a normal distribution, we used a univariate ANOVA to calculate the differences in the dependent variables (absolute and relative values) between the four groups (LWE vs. HWE vs. LWP vs. HWP), followed, if significant, by Bonferroni post hoc tests. In addition, we calculated the Hedge’s *g* effect size to determine the magnitude of the differences between groups and interpreted it based on the recommendations for highly trained individuals in resistance training: <0.25, trivial; 0.25–0.50, small; 0.50–1.00, moderate; >1.00, large [21]. Finally, by pooling the data of all groups, Pearson correlation coefficients with 95% CI were calculated to determine the relationships between the dependent variables. We interpreted the magnitude of correlation as: 0.00–0.10, negligible; 0.10–0.39, weak; 0.40–0.69, moderate; 0.70–0.89, strong; 0.90–1.00, very strong [22]. We set the alpha level at *p* < 0.05. We conducted the statistical analyses in Microsoft Office Excel (Microsoft Inc., Redmond, WA, USA) and SPSS v27 (SPSS Inc., Chicago, IL, USA).

## 3. Results

The results of normality and homogeneity of the variances can be found in the Supplementary Materials (Table S1). Table 2 shows the absolute and relative values of the handgrip and isometric lumbar strength, 1RM bench press, and 4RM leg press according to the competitive level and weight class. The univariate ANOVA showed differences between groups for the absolute 1RM bench press (*F*(3,17) = 9.32, *p* < 0.001), relative 1RM bench press (*F*(3,17) = 4.35, *p* = 0.02), and absolute isometric lumbar strength (*F*(3,17) = 4.25, *p* = 0.02). On the other hand, the univariate ANOVA revealed no differences between groups for the absolute 4RM leg press (*F*(3,17) = 1.76, *p* = 0.19), relative 4RM leg press (*F*(3,17) = 0.24, *p* = 0.87), relative isometric lumbar strength (*F*(3,17) = 1.00, *p* = 0.42), absolute handgrip strength left hand (*F*(3,17) = 0.10, *p* = 0.42), relative handgrip strength left hand (*F*(3,17) = 0.30, *p* = 0.83), absolute handgrip strength right hand (*F*(3,17) = 2.39, *p* = 0.10), and relative handgrip strength right hand (*F*(3,17) = 0.46, *p* = 0.71). Post hoc comparison tests showed large differences in the absolute 1RM bench press between the HWE and LWE groups (*p* = 0.01; *g* = 2.2). In addition, there were large differences between the HWE and LWP groups in the absolute (*p* < 0.001; *g* = 2.5) and relative 1RM bench press (*p* = 0.02; *g* = 1.7). There were no significant post hoc differences between groups (*p* > 0.05) for the remaining outcome variables. Table S2 shows the magnitude of differences between groups for each outcome.

Table 3 presents the correlation results with absolute and relative values. There was a moderate correlation between the 1RM bench press and isometric lumbar strength for the absolute (*r* = 0.67; *p* < 0.01) and relative values (*r* = 0.50; *p* = 0.02).

## 4. Discussion

### 4.1. Main Findings

The current research analyzed whether the maximum isometric and dynamic strength parameters could differentiate MMA athletes according to their competitive level and weight class. The results showed that the 1RM bench press and isometric lumbar strength could differentiate MMA athletes according to their competitive level and weight class. Therefore, the current study suggests that MMA athletes competing in higher weight classes and competitive levels might require higher upper-limb dynamic strength and isometric lumbar back strength levels than those competing in lower levels and weight classes. Nevertheless, given the small sample size in each group, these data should be considered preliminary, and future studies with a large sample size of professional and elite MMA athletes are warranted to refute or corroborate these observations.

### 4.2. Isometric Handgrip Strength

The dispute for controlling the opponent’s body is an efficient way of performing attack and defense techniques [23], and the handgrip strength is critical during these actions [12,17]. Indeed, Iermakov et al. [24] suggested the handgrip strength as a successful physical attribute in throws and grips to immobilize the opponent’s body. However, our results showed lower handgrip strength levels of MMA athletes than professional [15] and elite [25] judokas. Furthermore, the lightweight MMA athletes in our study showed lower values than experienced and amateur jiu-jitsu athletes [26] and international jiu-jitsu medalists [27]. As Bernardi et al. [27] suggested, the training and competition characteristics likely influence this result, and it is plausible to infer that this physical ability is not at the top of relevance for the MMA sport, as opposed to judo, jiu-jitsu, and even boxing.

The current study showed that both hands’ absolute and relative values of handgrip strength were higher for the HWP group than for the HWE group. However, there were no absolute and relative handgrip strength differences regardless of the competitive level and weight class. These results differ from Franchini et al. [17] with judokas, which observed that heavyweight athletes presented the highest relative values. This divergence between results could be because most professional athletes come from domain modalities where handgrip strength is a relevant factor for the success of their actions [28].

### 4.3. Isometric Lumbar Strength

Lumbar strength development is essential for combat modalities as a preventive factor for lower back pain, especially when the fighting strategy involves projections or actions to control the opponent on the ground [29,30]. Therefore, given that MMA includes high versatility of movements, and a high level of lumbar demand, strengthening this region through, for instance, back extension and deadlift exercises [10,31] is crucial for preventing injuries in combat athletes, especially before competitive phases [31]. Our data showed differences between groups on isometric lumbar strength, suggesting that heavyweight athletes present higher lumbar strength demands than lightweight athletes. Indeed, the heavyweight athletes in the present study presented higher lumbar strength values than regional-level MMA heavyweights [19] and Brazilian jiu-jitsu athletes [32]. In addition, it is essential to notice that the isometric lumbar strength was correlated with the 1RM bench press, suggesting that MMA athletes with high isometric lumbar strength values are more prone to present high 1RM bench press results and vice-versa. These results are probably due to the heavyweight athletes having presented higher back strength and 1RM bench press values than lightweight athletes. Therefore, strengthening isometric lumbar strength in MMA athletes is crucial for injury prevention and a distinguishing factor in athletes with the ambition to compete in higher weight classes.

### 4.4. Dynamic Bench Press Strength

Regarding the 1RM bench press results, the HWE athletes presented higher absolute 1RM values than the LWE and LWP athletes, suggesting that the maximum force production capacity in the bench press can discriminate between MMA athletes according to the competitive level and weight class. These results partially agree with previous research, showing that high-level MMA athletes tended to present higher 1RM bench press values than low-level athletes [14]. Similarly, previous findings observed that heavyweight athletes presented higher values than professional MMA athletes [15]. In addition, some research indicates that dynamic bench press strength is strongly associated with the maximal punching power in combat athletes [31] and punching velocity, especially in the rear hand [33]. Furthermore, when analyzing the influence of body weight, the HWE athletes showed higher values than the LWP athletes. Interestingly, the HWE also showed higher relative 1RM bench press values than those presented in previous studies with semi-professional and amateur MMA athletes classified as low-level (1.1 ± 0.2 kg·BW^−1^) and high-level competitors (1.2 ± 0.2 kg·BW^−1^) [14]. Therefore, these results collectively suggest that the 1RM bench press is a determinant test to incorporate into the testing routines of MMA athletes due to its ability to differentiate elite athletes according to weight class and competitive level.

### 4.5. Dynamic Leg Press Strength

As for the 4RM leg press data, although heavyweight athletes presented higher absolute values than lightweight athletes, there were no differences between groups. Therefore, these results suggest that the 4RM leg press test does not significantly discriminate between MMA athletes according to weight class and competitive level. Previous research observed that high-level MMA athletes showed higher 1RM back squat relative values than low-level MMA athletes (1.8 ± 0.2 kg·BW^−1^ vs. 1.6 ± 0.2 kg·BW^−1^, respectively) [14]. Based on these results, the authors suggested that MMA athletes should strive to optimize maximal lower-limb dynamic strength, since this attribute seems associated with competitive success [14]. Nevertheless, since we implemented a different exercise and a submaximal instead of a maximal test (1RM), these results cannot be directly compared. In addition, it is essential to notice that James et al. [14] conducted the study with semi-professional and amateur MMA athletes. Therefore, given the differences between experimental procedures, future studies with elite and professional MMA athletes should administer the 1RM back squat test and, if possible, the 1RM leg press test to understand whether lower-limb maximum dynamic strength is a discriminatory ability in athletes of different weight classes and competitive levels.

### 4.6. Limitations and Future Research

The current study presents several limitations we need to address. Firstly, the small sample size, which limits the statistical power and increases the type II error, does not enable us to generalize the results to other MMA athletes, and therefore they should be considered preliminary. Nevertheless, as previous Olympic coaches and researchers have mentioned, finding and recruiting a large and homogenous group of elite athletes of individual sports for research purposes is extraordinarily challenging and complex [34]. Therefore, given the lack of studies on professional and elite MMA athletes, the current findings should be considered another step towards a better understanding of maximal strength needs in MMA according to weight class and competitive level. Secondly, more strength tests assessing different muscle regions would be essential to understand their capacity to differentiate MMA athletes according to weight class and competitive level. In addition, the absence of the 1RM leg press or back squat test can also be considered a limitation since its administration, which requires more physical and mental effort than the 4RM test, would better represent their maximum lower-limb dynamic strength. However, we must note that we opted for the 4RM leg press test to avoid changing their testing routines, thus ensuring the reliability of data collection. Therefore, future studies with MMA athletes should include larger sample sizes of professional and elite athletes and a more comprehensive physical fitness test battery to understand what strength variables best discriminate MMA athletes according to weight class and competitive level.

## 5. Conclusions

The current study demonstrated that the 1RM bench press and isometric lumbar back strength were associated and could differentiate MMA athletes according to their competitive level and weight class. Specifically, heavyweight elite MMA athletes presented higher upper-limb dynamic strength and isometric lumbar strength than lightweight elite and professional athletes. Therefore, these data highlight the importance of MMA athletes optimizing upper-limb dynamic strength and isometric lumbar strength through strength training programs. A particular focus on heavy strength training might be required for lightweight MMA athletes, and a combination between heavy strength training and Olympic lifts and plyometrics can be an effective strategy for heavyweight MMA athletes.

## Figures and Tables

**Table 1:** Characteristics of the MMA athletes according to their competitive level and weight class.

	Age (y)		Height (m)		Body Weight (kg)	
	Mean ± SD	95% CI	Mean ± SD	95% CI	Mean ± SD	95% CI
Lightweight Professional (*n* = 9)	30.5 ± 6.2	27.8–33.2	1.73 ± 0.02	1.71–1.75	73.2 ± 3.5	70.9–75.5
Lightweight Elite (*n* = 4)	25.3 ± 1.9	24.5–26.1	1.72 ± 0.09	1.63–1.81	68.3 ± 3.8	64.6–72.1
Heavyweight Professional (*n* = 4)	28.3 ± 3.9	26.6–30.0	1.83 ± 0.05	1.78–1.87	89.9 ± 8.4	81.7–98.1
Heavyweight Elite (*n* = 4)	29.1 ± 6.0	26.5–31.7	1.79 ± 0.07	1.73–1.86	80.4 ± 1.5	78.9–81.8

Notes: SD: standard deviation; CI: confidence interval.

**Table 2:** Absolute and relative isometric and dynamic strength values according to the competitive level and weight class.

Variables	Lightweight Elite	Heavyweight Elite	Lightweight Professional	Heavyweight Professional
	Mean ± SD (95% CI)	Mean ± SD (95% CI)	Mean ± SD (95% CI)	Mean ± SD (95% CI)
HGS-R (kgf)	36.0 ± 5.9 (30–42)	45.3 ± 7.1 (38–52)	36.1 ± 7.8 (31–41)	44.5 ± 7.5 (37–52)
HGS-L (kgf)	39.5 ± 8.1 (32–47)	41.8 ± 7.2 (35–49)	38.3 ± 7.4 (33–43)	46.0 ± 8.2 (38–54)
Rel HGS-R (kgf·BW^−1^)	0.5 ± 0.1 (0.4–0.6)	0.6 ± 0.1 (0.5–0.6)	0.5 ± 0.1 (0.4–0.6)	0.5 ± 0.1 (0.4–0.6)
Rel HGS-L (kgf·BW^−1^)	0.6 ± 0.1 (0.5–0.7)	0.5 ± 0.1 (0.4–0.6)	0.5 ± 0.1 (0.5–0.6)	0.5 ± 0.1 (0.4–0.6)
HGS-R&L (kgf)	75.5 ± 12.9 (63–88)	87.0 ± 12.1 (75–99)	74.4 ± 14.6 (65–84)	90.5 ± 10.5 (80–101)
Rel HGS-R&L (kgf·BW^−1^)	1.1 ± 0.2 (0.9–1.3)	1.1 ± 0.1 (0.9–1.2)	1.0 ± 0.2 (0.9–1.2)	1.0 ± 0.2 (0.8–1.2)
ILS (kgf)	158.0 ± 3.9 (154–162)	185.3 ± 10.4 (175–195)	158.9 ± 15.6 (149–169)	184.0 ± 26.9 (158–210)
Rel ILS (kgf·BW^−1^)	2.3 ± 0.1 (2.2–2.5)	2.3 ± 0.1 (2.2–2.4)	2.2 ± 0.3 (2.0–2.4)	2.1 ± 0.3 (1.8–2.3)
1RM-BP (kg)	81.0 ± 10.0 (71–91)	117.0 ± 17.8 (100–134) *†	76.7 ± 13.7 (68–86)	100.0 ± 12.1 (88–112)
Rel 1RM-BP (kg·BW^−1^)	1.2 ± 0.1 (1.0–1.3)	1.5 ± 0.2 (1.2–1.7) †	1.1 ± 0.2 (0.9–1.2)	1.1 ± 0.1 (1.1–1.2)
4RM-LP (kg)	600.0 ± 119.4 (483–717)	642.5 ± 109.0 (536–749)	602.2 ± 100.6 (537–668)	740.0 ± 100.3 (642–838)
Rel 4RM-LP (kg·BW^−1^)	8.9 ± 2.4 (6.6–11.2)	8.0 ± 1.4 (6.6–9.4)	8.2 ± 1.4 (7.3–9.1)	8.2 ± 0.9 (7.3–9.1)

Notes: * Significant differences (*p* < 0.05) between the heavyweight and lightweight elite groups. † Significant differences (*p* < 0.001) between the heavyweight elite and lightweight professional groups. 1RM-BP: one-repetition maximum bench press; 4RM-LP: four-repetition maximum leg press; HGS-R: handgrip strength right hand; HGS-L: handgrip strength left hand; HGS-R&L: handgrip strength right and left hands; ISL: isometric lumbar strength; Rel: relative.

**Table 3:** Pearson correlation results with absolute and relative values (normalized to body weight).

Variables	*r*	*p*
4RM-LP and 1RM-BP	0.19	0.41
4RM-LP and Lumbar Strength	0.17	0.61
4RM-LP and HGS-R&L	0.23	0.32
1RM-BP and Lumbar Strength	0.67	<0.001
1RM-BP and HGS-R&L	0.24	0.31
Lumbar Strength and HGS-R&L	0.18	0.43
Rel 4RM-LP and Rel 1RM BP	0.02	0.92
Rel 4RM-LP and Rel Lumbar Strength	0.09	0.72
Rel 4RM-LP and Rel HGS-R&L	0.16	0.49
Rel 1RM-BP and Rel Lumbar Strength	0.50	0.02
Rel 1RM-BP and Rel HGS-R&L	0.11	0.64
Rel Lumbar Strength and Rel HGS-R&L	0.16	0.49

Notes: 1RM-BP: one-repetition maximum bench press; 4RM-LP: four-repetition maximum leg press; HGS-R&L: handgrip strength right and left hands; Rel: relative.

## Data Availability

Data available on a reasonable request.

## Supplementary Materials

The following supporting information can be downloaded at: https://www.mdpi.com/article/10.3390/ijerph19148741/s1, Table S1: Shapiro–Wilk and Levene’s tests data; Table S2: Hedge’s *g* effect size for the comparison between groups.
