# Supplementary material for: Maximum Isometric and Dynamic Strength of Mixed Martial Arts Athletes According to Weight Class and Competitive Level
Source: Int J Environ Res Public Health. 2022 Jul 18;19(14):8741. doi: 10.3390/ijerph19148741 (PMC9323058; doi:10.3390/ijerph19148741)
Supplement: Supplementary file 1 [file ijerph-19-08741-s001.zip › ijerph-1807855-supplementary.pdf]

Supplementary Materials

Maximum isometric and dynamic strength of Mixed Martial Arts athletes according to weight class and competitive level

Table S1. Shapiro-Wilk and Levene's tests data.

Table S2. Hedge’s g effect size for the comparison between groups.

Table S1. Shapiro-Wilk and Levene's tests data.

Variable	Shapiro-Wilk (sig.)	Levene test (sig.)
4RM-LP	0.114	0.989
1RM-BP	0.728	0.652
ILS	0.450	0.111
HGS-L	0.420	0.969
HGS-R	0.779	0.916
HGS-R&L	0.493	0.922
4RM-LP Rel	0.137	0.436
1RM-BP Rel	0.789	0.212
ILS Rel	0.141	0.383
HGS-L Rel	0.134	0.910
HGS-R Rel	0.370	0.912
HGS-R&L Rel	0.454	0.846

Notes: 1RM-BP: one-repetition maximum bench press; 4RM-LP: four-repetition maximum leg press; ILS: isometric lumbar strength; HGS-R: handgrip strength right hand; HGS-L: handgrip strength left hand; HGS-R&L: handgrip strength right and left hands; Rel: relative.

Table S2. Hedge's g effect size for the comparison between groups.

	Hedge's g											
	**4RM-LP**	**1RM-BP**	**ILS**	**HGS-L**	**HGS-R**	**HGS-R&L**	**4RM-LP Rel**	**1RM-BP Rel**	**ILS Rel**	**HGS-L Rel**	**HGS-R Rel**	**HGS-R&L Rel**
HWE vs LWE	0.32	2.17	3.02	0.26	1.23	0.80	-0.39	1.26	-0.10	-0.50	0.38	-0.12
HWE vs LWP	0.36	2.52	1.71	0.43	1.12	0.84	-0.16	1.71	0.43	-0.05	0.64	0.31
HWE vs HWP	-0.81	0.97	0.05	-0.48	0.09	-0.27	-0.16	1.92	1.02	0.02	0.50	0.31
LWP vs HWP	1.19	1.57	0.99	0.85	0.95	1.10	-0.01	0.30	-0.38	-0.07	0.07	0.00
LWP vs LWE	0.02	-0.31	0.07	-0.13	0.01	-0.07	-0.29	-0.61	-0.50	-0.42	-0.31	-0.39
LWE vs HWP	-1.10	-1.49	-1.17	-0.70	-1.09	-1.11	0.32	0.60	1.01	0.47	0.20	0.40
HWE vs LWE	0.32	2.17	3.02	0.26	1.23	0.80	-0.39	1.26	-0.10	-0.50	0.38	-0.12

Notes: 1RM-BP: one-repetition maximum bench press; 4RM-LP: four-repetition maximum leg press; ILS: isometric lumbar strength; HGS-R: handgrip strength right hand; HGS-L: handgrip strength left hand; HGS-R&L: handgrip strength right and left hands; Rel: relative; HWE: heavyweight elite; LWE: lightweight elite; HWP: heavyweight professional; LWP: lightweight professional.
